# Neutrophil extracellular traps (NETs) exacerbate severity of infant sepsis

**DOI:** 10.1186/s13054-019-2407-8

**Published:** 2019-04-08

**Authors:** David F. Colón, Carlos W. Wanderley, Marcelo Franchin, Camila M. Silva, Carlos H. Hiroki, Fernanda V. S. Castanheira, Paula B. Donate, Alexandre H. Lopes, Leila C. Volpon, Silvia K. Kavaguti, Vanessa F. Borges, Cesar A. Speck-Hernandez, Fernando Ramalho, Ana P. Carlotti, Fabio Carmona, Jose C. Alves-Filho, Foo Y. Liew, Fernando Q. Cunha

**Affiliations:** 1 Department of Biochemistry and Immunology, School of Medicine of Ribeirão Preto, University of São Paulo, Ribeirão Preto, 14049-900 SP Brazil; 2 Department of Physiology and Pharmacology, Federal University of Ceará, Fortaleza, 60020-181 CE Brazil; 3 Department of Pharmacology, University of Campinas, Campinas, 13083-970 SP Brazil; 4 Department of Pharmacology, School of Medicine of Ribeirão Preto, University of São Paulo, Ribeirão Preto, SP 14049-900 Brazil; 5 Department of Pathology, School of Medicine of Ribeirão Preto, University of São Paulo, Ribeirão Preto, 14049-900 SP Brazil; 6 Pediatrics, School of Medicine of Ribeirão Preto, University of São Paulo, Ribeirão Preto, 14049-900 SP Brazil; 7 Division of Immunology, Infection and Inflammation, Glasgow Biomedical Research Centre, University of Glasgow, Glasgow, G128QQ UK; 8 School of Biology and Basic Medical Science, Soochow University, Suzhou, 215006 JS China

**Keywords:** Infant, Sepsis, Susceptibility, Neutrophil extracellular traps, *Padi4*

## Abstract

**Background:**

Neutrophil extracellular traps (NETs) are innate defense mechanisms that are also implicated in the pathogenesis of organ dysfunction. However, the role of NETs in pediatric sepsis is unknown.

**Methods:**

Infant (2 weeks old) and adult (6 weeks old) mice were submitted to sepsis by intraperitoneal (i.p.) injection of bacteria suspension or lipopolysaccharide (LPS). Neutrophil infiltration, bacteremia, organ injury, and concentrations of cytokine, NETs, and DNase in the plasma were measured. Production of reactive oxygen and nitrogen species and release of NETs by neutrophils were also evaluated. To investigate the functional role of NETs, mice undergoing sepsis were treated with antibiotic plus rhDNase and the survival, organ injury, and levels of inflammatory markers and NETs were determined. Blood samples from pediatric and adult sepsis patients were collected and the concentrations of NETs measured.

**Results:**

Infant C57BL/6 mice subjected to sepsis or LPS-induced endotoxemia produced significantly higher levels of NETs than the adult mice. Moreover, compared to that of the adult mice, this outcome was accompanied by increased organ injury and production of inflammatory cytokines. The increased NETs were associated with elevated expression of *Padi4* and histone H3 citrullination in the neutrophils. Furthermore, treatment of infant septic mice with rhDNase or a PAD-4 inhibitor markedly attenuated sepsis. Importantly, pediatric septic patients had high levels of NETs, and the severity of pediatric sepsis was positively correlated with the level of NETs.

**Conclusion:**

This study reveals a hitherto unrecognized mechanism of pediatric sepsis susceptibility and suggests that NETs represents a potential target to improve clinical outcomes of sepsis.

**Electronic supplementary material:**

The online version of this article (10.1186/s13054-019-2407-8) contains supplementary material, which is available to authorized users.

## Background

Sepsis is a life-threatening multi-organ dysfunction caused by dysregulated host response to infection with unmet clinical needs [1, 2]. Infants are particularly susceptible to sepsis and have a higher risk of long-term complications and mortality [3–5]. Despite this serious impact, there remains scant literature on the immune pathological mechanisms and specific targets for treatment of pediatric sepsis.

The “immature status” of infant immunological systems has frequently been attributed to the high sepsis susceptibility in these groups of patients [6–8]. However, one hallmark of sepsis in newborns is an extremely rapid course of hyper-inflammatory immune response [9]. Moreover, cord blood cells and monocytes from neonates cultured with ligands of toll-like receptor (TLR) released higher levels of proinflammatory cytokines than that produced by similarly cultured adult blood cells [10, 11]. Furthermore, dendritic cells from infants produced high levels of IL-23 and IL-12 comparable to that of adult cells when stimulated with lipopolysaccharide (LPS) in vitro [12]. Additionally, during endotoxemia, neonatal monocytic myeloid-derived suppressor cells change to inflammatory monocyte status, contributing to hyper-inflammatory responses and fatal septic course [13]. Thus, the pathogen-associated molecular pattern (PAMP) recognition and assembly of the innate immune response are not compromised in infants.

Effective control of the multiplication of microorganisms by innate immune cells is the essential event for limiting the infection [14]. Neutrophils are the key players, providing the first line of host defense through rapid recruitment to the sites of infection, where they phagocytize the pathogens and produce microbicidal mediators, including free radicals [15]. However, severe sepsis can induce a “dysregulation” of neutrophil migration to infectious foci; instead, neutrophils accumulate in vital organs, such as the lung, kidney, and liver [16]. This phenomenon results in a reduction of the host’s ability to control the infection, but increases tissue damage with the development of organ dysfunction [17]. However, the mechanism by which the infiltrating neutrophils damage these vital organs during severe sepsis is obscure.

Recently, a new neutrophil effector function, neutrophil extracellular traps (NETs), has been reported [18]. NETs are an important part of innate immune defense and are involved in the control of infections [18–20]. NETs are an extracellular DNA matrix, also containing granule proteins and histones, released by neutrophils to degrade virulence factors and to kill bacteria [18]. NETs are primarily considered a protective mechanism against a broad range of microorganisms [18, 21, 22]; however, increasing evidence has shown that NETs have also been implicated in the pathogenesis of organ dysfunction [23–25].

Reports on the role of NETs in infant sepsis physiopathology are sparse. The aim of our study was to investigate the role of NETs in infant sepsis susceptibility (mortality) induced experimentally by polymicrobial infection and by LPS. We report here that compared to that produced by adult septic mice, neutrophils from infant septic mice produced significantly higher concentrations of NETs. Furthermore, degradation of NETs by rhDNase or a PAD-4 inhibitor in vivo markedly increased the survival rate of severe infant sepsis in mice. The increased NET production is also associated with the clinical indicator of pediatric sepsis severity (PRISM score). Our results suggest that targeting NETs represents a potential therapeutic option in treating infant sepsis.

## Methods

### Animals

Infant (2 weeks old) and adult (6 weeks old) C57BL/6 mice (wild-type, WT) were obtained from the animal facility of the Ribeirao Preto Medical School of the University of São Paulo, São Paulo, Brazil. A total of 828 male mice (427 infants and 356 adults) were randomly distributed among experiments, and no animal was excluded during our study (see Additional file 1: Figure S1). The animals were housed under standard conditions and received water and food ad libitum. Mice were housed in barrier cages under controlled environmental conditions (12/12 h of light/dark cycle, 55% ± 5% humidity, 23 °C).

### Patients

Peripheral blood samples were collected from 26 septic patients (15 pediatrics and 11 adults), who were prospectively enrolled in the study within the first 24 h of admission to the emergency department of a tertiary-care university hospital of Ribeirão Preto. All patients presented clinical or laboratory variables that fulfilled the criteria for sepsis [26]. Thirteen healthy volunteers (7 pediatrics and 6 adults) were recruited as controls. Pediatric severity was evaluated by the Pediatric Risk of Mortality (PRISM) score and organ dysfunction by the Pediatric Logistic Organ Dysfunction (PELOD) score [27, 28]. Total inotropic support was estimated by a modified inotropic score, calculated as follows: doses of dopamine + dobutamine + milrinone × 10 + epinephrine × 100 + norepinephrine × 100 [29]. The values of PRISM, PELOD, and inotropic scores were not normally distributed. Therefore, they were log-transformed to approximate a normal distribution, which would render more reliable correlation results, eliminating any undue influence of outliers. We calculated Pearson’s *r* coefficients with corresponding *p* values for all pairs of correlations between NETs and PRISM, PELOD, or inotropic scores.

### Bacterial culture

The cecal content of an adult C57BL/6 mouse was isolated, filtered through sterile gauze, and grown in brain heart infusion (BD Diagnostic Systems, Sparks, USA) for 5 days at 37 °C. Bacteria grown in this culture were washed two times with PBS, lyophilized, and frozen on aliquots. One vial of bacteria was thawed and grown in a brain heart infusion (BHI) medium, 37 °C for 20 h before each experiment. After two rounds of washing to remove culture medium, bacteria were suspended on sterile saline 0.9% and the quantity of bacteria was assessed by absorbance at 600 nm using a spectrophotometer (Molecular Devices, Sunnyvale, USA).

### Experimental design

The study objective was to establish an experimental model of pediatric sepsis that mimics the high susceptibility of pediatric patients to sepsis compared to adults. Infant (2 weeks old) and adult (6 weeks old) mice were injected intraperitoneally with saline 0.9% (Sham) or a cecal bacterial suspension (4 × 10^7^ to 4 × 10^8^ colony-forming unit (CFU)/cavity). Survival was recorded daily, and serum biomarkers for organ functions were assayed at regular intervals. We also studied neutrophil functions in vitro and in vivo.

### Sepsis and endotoxemia models

Infant and adult mice received an intraperitoneal injection of 4 × 10^7^ to 4 × 10^8^ CFU/cavity bacteria. The survival rate was recorded daily for 5 days, and at the end of this period, the remaining mice were euthanized by ketamine/xylazine overdose (> 100 mg/kg, s.c., União Quimica, Brazil) followed by cervical dislocation. In the endotoxemia model, mice received LPS (30 or 35 mg/kg, i.p.). After dose standardization, a dose of 2 × 10^8^ CFU/mouse of bacteria was used for sepsis induction for all subsequent experiments. In experiments performed plus antibiotic, septic mice received an i.p. injection of ertapenem sodium (Merck Research Laboratory), 30 mg kg^− 1^ to adult mice and 15 mg kg^− 1^ to infant mice, beginning 6 h after sepsis and then every 12 h up to day 3.

### Bacterial counts

Bacterial counts were determined 6 h after infection, as previously described [30]. Briefly, peritoneal exudate and blood samples were collected, serially diluted, plated on Muller-Hinton agar dishes (Difco Laboratories), and incubated at 37 °C for 18 h, and CFU per milliliter were recorded.

### Blood urea nitrogen (BUN) and creatinine levels, glutamic oxaloacetate transaminase (GOT) and creatine kinase-MB isoenzyme (CK-MB) activity

Animals were euthanized 6 or 18 h after infection or LPS injection, and blood samples were collected to measure renal, hepatic, and cardiac dysfunctions, as assessed by BUN, GOT, and CK-MB levels, respectively. Additionally, 3, 6, and 9 h after sepsis induction, the blood samples were collected and serum creatinine levels were assessed. The assays were performed with a commercial kit (Labtest, Brazil).

### Myeloperoxidase assay

Tissue myeloperoxidase (MPO) activity was used as a biochemical index of neutrophil infiltration into the lungs, as previously described [17, 31].

### Cytokine assays

Cytokine concentrations were measured by ELISA, using antibodies from R&D Systems according to the manufacturer’s instructions. The optical density of the individual samples was measured at 450 nm using a spectrophotometer (Spectra Max-250, Molecular Devices, Sunnyvale, CA, USA), as previously described [32].

### Neutrophil purification

Bone marrow (BM) and circulating neutrophils were isolated by Percoll density gradient, as previously described [17]. Briefly, two different gradients were prepared in a 15-ml polystyrene tube with 3 ml each (72% and 65% Percoll solutions). After centrifugation at 1200×*g* for 30 min at 25 °C, the cell layer at the 72% upper interface was collected as the neutrophil fraction. Erythrocytes were removed by lysis (NH_4_), and the remaining neutrophil fractions were washed twice in HANKS [33]. The pelleted cells were resuspended in 1 ml of RPMI 1640 medium (Sigma Chemical Co., St Louis, USA), and the number of neutrophils was determined by Neubauer chamber counting and purity by Wright-Giemsa staining. BM neutrophils were then incubated with phorbol myristate acetate (PMA) or LPS for NET measurement, and circulating neutrophils were used for determining *Padi4* expression. Although neutrophils can be easily isolated in large numbers from human blood, this method is suboptimal in mice due to the limited volume of blood that precludes isolation of sufficient neutrophils for functional studies. In addition, although the yield of thioglycollate-elicited cells from the peritoneal cavity is greater compared to that of mouse blood, the purity of neutrophils in the inflammatory peritoneal lavage varies between 60 and 90%, and the isolated neutrophils exhibit an activated phenotype [34, 35]. Instead, the bone marrow is a convenient reservoir for harvesting large numbers of either un-stimulated or activated neutrophil, which can then be used for downstream functional studies [34, 36–38].

### Nitric oxide production

Neutrophils (2 × 10^5^ cells) were incubated with RPMI 1640 medium containing LPS (10 ng/ml) for 0, 3, and 12 h at 37 °C in a 5% CO_2_. For macrophages, 2 × 10^5^ cells were incubated with RPMI 1640 medium containing LPS (10 ng/ml) for 4 h. The total amount of nitrite in the culture supernatant was determined by the Griess method.

### Total reactive oxygen species production

Bone marrow neutrophils or peritoneal macrophages (0.25 × 10^6^/ml) of mice were incubated with Luminol (125 mM) and opsonized zymozan particles (20 mg/ml) or PMA (25 nM). Total oxygen species production was measured on a FlexStation 3 Benchtop Multi-Mode Microplate Reader.

### Treatment with rhDNase

Mice were injected i.p. with saline or rhDNase (10 mg/kg, sc.) 10 min and 4 h after LPS injection or sepsis induction and then every 12 h thereafter, as previously reported [39]. Expression of *Padi4* mRNA and H3-histone citrullination in the lung and circulating neutrophils was determined 18 h after LPS injection.

### Treatment with cl-Amidine

Mice were injected with Cl-Amidine (50 mg/kg, ip.), an inhibitor of PAD4, 1 h prior to sepsis induction, as previously reported [40], with or without antibiotic treatment (i.p. injection of ertapenem sodium, 30 mg kg^− 1^ to adult mice and 15 mg kg^− 1^ to infant mice, beginning 6 h after sepsis and then every 12 h up to day 3).

### NET (cfDNA/MPO) quantification

This procedure was performed as previously described [41]. Briefly, an antibody bound to the 96-well clear-bottom black plate captured the enzyme MPO (5 μg/ml; sc-16128-R, Santa Cruz Biotechnology), and the amount of DNA bound to the enzyme was quantified using the Quant-iT™ PicoGreen® kit (Invitrogen) according to the manufacturer’s instructions. The fluorescence intensity (excitation at 488 nm and emission at 525 nm wavelength) was quantified by a FlexStation 3 Microplate Reader (Molecular Devices, CA, USA).

### DNase activity assay

Animals were euthanized 6 h after infection, and blood samples were collected to evaluate the plasma DNase activity. Briefly, isolated plasma from adult and infant septic mice were incubated with λDNA (1 μg/ml) for 3 h at 37 °C in a 5% CO_2_. The amount of λDNA digested was quantified using the Quant-iT™ PicoGreen® kit (Invitrogen) according to the manufacturer’s instructions. The fluorescence intensity (excitation at 488 nm and emission at 525 nm wavelength) was quantified by a FlexStation 3 Microplate Reader (Molecular Devices, CA, USA). The plasma DNase activity was determined by the Delta of the fluorescence of the control (DNA + picogreen) vs. DNA plus plasma.

### Phagocytosis of NETs by macrophages

Macrophages were obtained from the peritoneal cavity of mice [42]. Adult and infant mice were injected i.p. with 2 mL and 1 mL of sterile 3% thioglycollate medium, respectively. After 3 days, macrophages were obtained by washing the peritoneal cavity with 2.5 ml of RPMI 1640 medium (Sigma). Cells were seeded at 3 × 10^4^ cells/well in 150 μL culture medium with 10% fetal bovine serum. The cells were marked with Hoechst (5 μg/mL).

The NET suspension was obtained according to Najmeh et al. [43]. Briefly, isolated neutrophils from adult and infant mice were stimulated with 50 nM of PMA and incubated for 4 h at 37 °C 5% CO_2_. After 4 h of stimulation, adhered NETs and neutrophils were removed using cold PBS. The solution obtained was centrifuged for 10 min at 450×*g* at 4 °C; the remaining cells were pelleted at the bottom, leaving a cell-free NET-rich supernatant. The supernatant was then centrifuged for 10 min at 18,000×*g* at 4 °C, forming a NET pellet, which was resuspended with culture medium.

NETs were prepared with 250 nM Sytox green and added on macrophages (final concentration of 250 ng/mL NETs) or culture medium (as negative control) and incubated for 4 h. Fluorescent and bright-field images (356.4 × 356.4 μm) were acquired using an ImageXpress Micro XLS WideField High-Content Analysis System with a 0.60 numerical aperture × 40 objective. Fluorescent images were acquired using a DAPI filter with excitation 377/50 nm and emission 447/60 nm, with an exposure time of 100 ms and a FITC filter with excitation and emission wavelengths of 482/35 nm and 536/40, with an exposure time of 10 ms. After acquisition, the images were quantified as NETs (DNA/MPO) in the supernatant, using the Quant-iT™ PicoGreen kit (Invitrogen) as previously described [41], by a fluorescence reader (FlexStation Microplate Reader, Molecular Devices, CA, USA).

### Immunofluorescence of NETs

The tissue segment was embedded in optimum cutting temperature, and sections (15 μm) were blocked and incubated with primary antibodies against Histone H3 citrulline R17+R2+R8 (1:1000; ab5103, Abcam) and then stained with conjugated secondary antibodies. Images and analysis were performed using a fluorescence microscope (Leica DMI6000B). For immunostaining, neutrophils were attached on slides coated with poly-d-lysine (Sigma) and stimulated with PMA (100 ng/ml) or medium. The slides were then fixed with 4% paraformaldehyde and stained with DAPI (P36935, Molecular Probes), anti-MPO (1:50; sc-16128-R, Santa Cruz Biotechnology), and anti-histone H4 (1:200, sc-25260, Santa Cruz Biotechnology) antibodies, followed by anti-rabbit Alexa Fluor 594 (1:100; Molecular Probes) or anti-mouse Alexa Fluor 488 (1:400; Molecular Probes). Confocal images were taken with a Leica TCS SP5-AOBS microscope (Leica Microsystems, Mannheim, Germany). Epifluorescence images were taken with a Zeiss Axioplan. Permeabilizing agents were not used for exclusive extracellular labeling. The percentage of NETs was determined from six non-overlapping fields per well and the average was from triplicates for each condition in every experiment. All analysis was performed blinded to treatment conditions.

### Gene expression by real-time PCR

Total RNA from the lung was extracted using TRIZOL reagent (Invitrogen) according to the manufacturer’s instructions. For neutrophils purified from the blood, total RNA was extracted using the RNeasy Mini Kit (Qiagen). Total RNA (2 μg for lung tissue and 400 ng for neutrophils) was reverse-transcribed using high-capacity cDNA RT Kit (Applied Biosystem). Briefly, cDNA was used as the template for qPCR of genes of interest (SYBR Green method) using a ViiA7 System (Applied Biosystems). Target gene expression was calculated using the comparative method for relative quantification after normalization to *Gapdh* gene expression. Real-time PCR was performed using the following primers: *Pad4,* F-TGACCAATGGATGCAGGACG / R-CTCTGTCCCTCGGGGAGTC and *Gapdh,* F- GGGTGTGAACCACGAGAAAT / R-CCTTCCACAATGCCAAAGTT.

### Western blot analysis

Mice were terminally anesthetized and the lungs collected. Samples were homogenized in a lysis buffer containing a mixture of proteinase inhibitors (Tris-HCl 50 mM, pH 7.4; NP-40 1%; Na-deoxycholate 0.25%; NaCl 150 mM; EDTA 1 mM; PMSF 1 mM; aprotinin, leupeptin, and pepstatin 1 μg/ml). Proteins were separated by SDS-polyacrylamide gel electrophoresis and trans-blotted onto nitrocellulose membranes (Amersham Pharmacia Biotech). The membranes were blocked with 10% dry milk and incubated overnight at 4 °C with rabbit polyclonal antibody against Histone H3 citrulline R17+R2+R8 (1:200; ab5103, Abcam). The membranes were incubated with secondary antibody (Jackson ImmunoResearch). Immunodetection was performed using an enhanced chemiluminescence light-detecting kit (Amersham Pharmacia, Biotech) for 1 min. The mouse monoclonal antibody against β-actin (1:5000; Sigma Aldrich) was used for loading controls. Optical densitometry was measured following normalization to the control (house-keeping gene) using Scientific Imaging Systems (Image labTM 3.0 software, Biorad Laboratories, Hercules, CA, USA).

### Histological examination

Mice were euthanized 6 h or 18 h after sepsis or LPS-induced endotoxemia. Lung and liver tissues were harvested and fixed in 4% buffered formalin and embedded in paraffin blocks. Sections (5 μm) were then stained with hematoxylin and eosin for histological examination. Leukocyte infiltration, edema, vascular congestion, collagen deposition, and steatosis were evaluated in a blinded fashion.

### Statistical analysis

The data (except for the survival curves) are reported as the mean ± standard error of the mean (SEM) of values obtained from at least two independent experiments. The means of different treatments were compared by analysis of variance (ANOVA) followed by Bonferroni’s Student’s *t* test for unpaired values. Bacterial counts were analyzed by the Mann-Whitney *U* test. The survival rate was expressed as the percentage of live animals, and the Mantel-Cox log-rank test was used to determine differences between survival curves. Spearman’s rank correlation coefficient (*σ*) was calculated to describe correlations between plasma cfDNA/NET concentrations and clinical variables. *P* < 0.05 was considered significant. All statistical analyses were performed using GraphPad Prism version 5.00 for Windows (GraphPad Software, USA).

## Results

### Infant mice are more susceptible than adult mice to sepsis

**Fig. 1:**
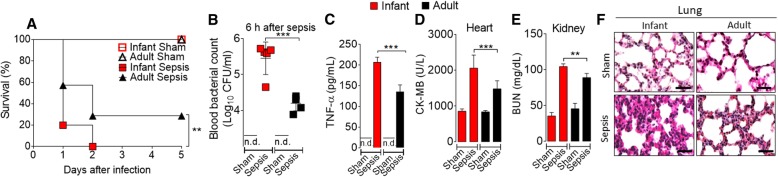
Infant mice are more susceptible than adult mice to sepsis. **a** Infant and adult mice were injected with microbial suspension (2 × 10^8^ CFU/cavity), and the survival was recorded for 5 days. **b** Bacterial counts in the blood. **c** Serum concentration of TNF-α was determined by ELISA 6 h after sepsis induction. The enzymatic activity of CK-MB (measure of heart damage) (**d**) and BUN (measure of kidney damage) (**e**) in the serum were determined. **f** Representative histology of lung sections is shown, magnification ×40. Bars = 100 μm. Data are mean ± SEM, *n* = 5–6 per group and are representative of two to three independent experiments. **p < 0.01, ***p < 0.001 (**a**, Mantel-Cox log-rank test; **b**–**e** one-way ANOVA, Bonferroni’s), n.d. = not detectable

To assess the susceptibility of infant mice to sepsis, infant (2 weeks old) and adult (6 weeks old) mice were submitted to sepsis by intraperitoneal (i.p.) injection of bacteria suspension (the dose of bacteria was estimated by OD600, Additional file 2: Figure S2A) or LPS. Infant mice developed a significantly higher mortality rate than the adult mice when injected with the same number of bacteria or the same bacterial load/kg body weight (Fig. 1a and Additional file 2: Figure S2B, C). Thus, for all subsequent experiments, a dose of 2 × 10^8^ CFU/mouse of bacteria was used to induce sepsis. The infant septic mice also showed higher bacteria counts in the blood and in the peritoneal exudate (Fig. 1b, Additional file 3: Figure S3A, B) and elevated levels of serum TNF-α, IL-6, and IL-1β (Fig. 1c and Additional file 3: Figure S3C, D) than in the adult septic mice. The infant mice exhibited more severe damage in the heart (creatine kinase MB isoenzyme, CK-MB, level) and kidney (blood urea nitrogen, BUN, level and creatinine), accompanied by vascular hypo-responsiveness to vasoconstrictors (Fig. 1d, e and Additional file 3: Figure S3E, F, Additional file 4. Table S1).

Compared to that of the adult septic mice, histological analysis also showed pulmonary vascular congestion and more severe edema and steatosis in the liver in the infant septic mice (Fig. 1f and Additional file 3: Figure S3G). Furthermore, compared to that of the adult mice, there was markedly increased neutrophil infiltration in the lungs of infant mice (Additional file 3: Figure S3H). We also compared the susceptibility of mice to LPS-induced shock. Infant mice sustained a markedly higher mortality rate than the adult mice when injected with 30 or 35 mg/kg body weight of LPS, accompanied by an elevated level of serum TNF-α (Additional file 3: Figure S3I, J and Additional file 2: Figure S2D). Notably, infant peritoneal macrophages stimulated with LPS in vitro produced higher levels of proinflammatory cytokines (TNF-α and IL-6) than the cells from adult mice (Additional file 3: Figure S3K, M). Collectively, these results demonstrate that infant mice are more susceptible to sepsis and LPS-induced endotoxemia with more severe multi-organ damage.

### Infant septic mice release higher amounts of NETs than adult mice

**Fig. 2:**
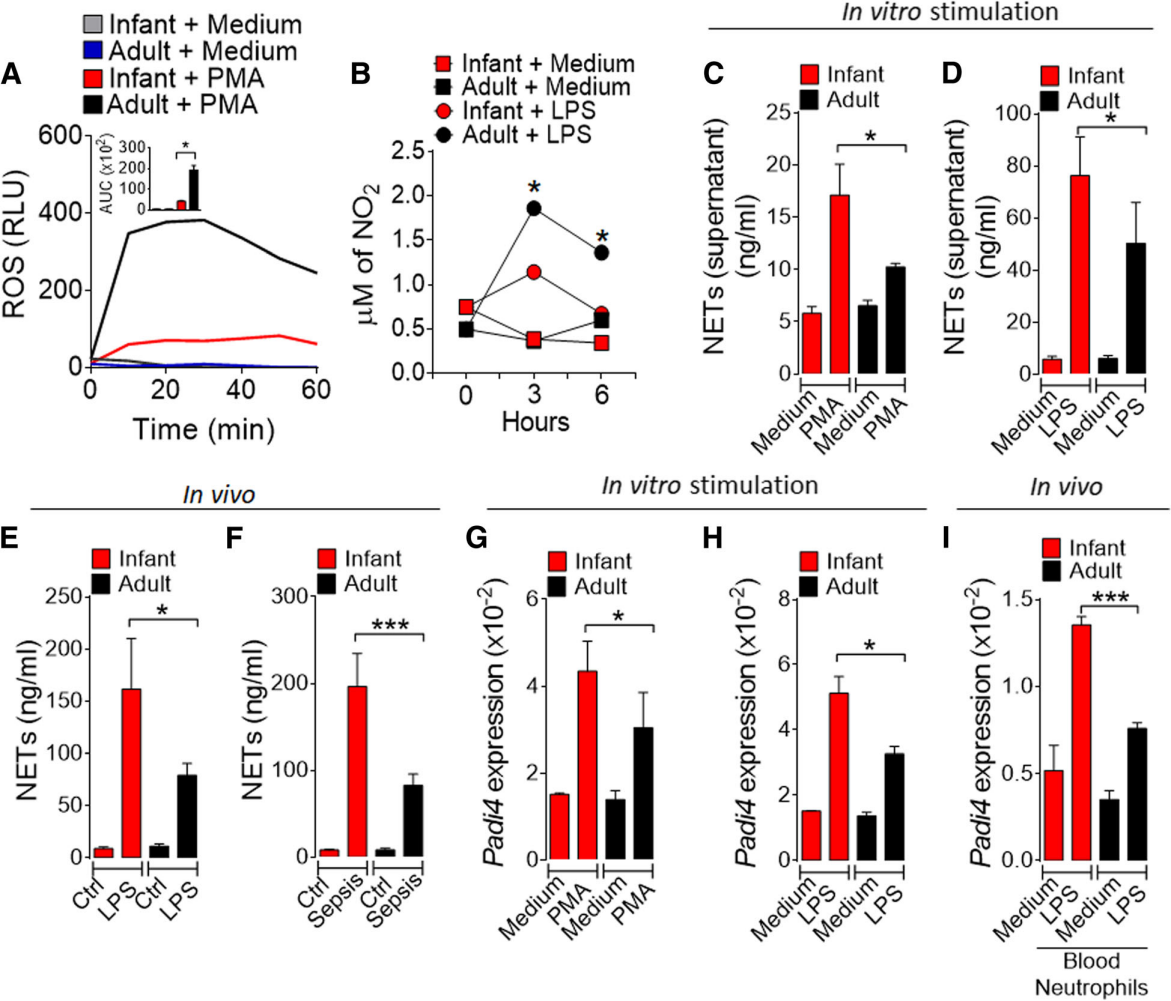
Infant mice release more NETs than adult mice. **a** Bone marrow-isolated neutrophils from mice were cultured with PMA and the production of ROS determined. **b** Neutrophils were cultured with LPS (10 ng/ml) for 0, 3, and 6 h and the production of nitric oxide measured. **c**, **d** Neutrophils were stimulated in vitro with PMA (100 ng/ml) (**c**) or LPS (500 ng/ml) (**d**) for 4 h. Concentrations of cfDNA/NETs (NETs) in the culture supernatant were determined. Mice were injected with LPS (**e**) or microbial suspension (sepsis) (**f**). NET concentration in the serum was determined 6 h or 18 h later, respectively. Neutrophils were cultured with PMA (**g**) or LPS (**h**) for 4 h and the expression of *Padi4* mRNA in the cells were determined by qPCR. **i** Mice were injected with LPS and neutrophils in the peripheral blood collected 18 h later and the expression of *Padi4* mRNA was determined. Ctr = control group, Sep = sepsis group. Data are the mean ± SEM, *n* = 5–6 per group and representative of two experiments, *p < 0.05, **p < 0.01, ***p < 0.001 (one-way ANOVA, Bonferroni’s)

We then investigated the mechanism associated with the multi-organ injury in the infant septic mice. Neutrophil infiltration is a key mediator of organ dysfunction through the production of reactive oxygen and nitrogen species (ROS and NOS) in the vital organs during sepsis [17, 44, 45]. We therefore investigated the relative levels of ROS and NO produced by bone marrow neutrophils isolated from infant and adult mice in response to activation by zymogen or LPS in vitro. Contra-intuitively, neutrophils from infant mice produced significantly less ROS and NO (Fig. 2a, b; Additional file 5: Figure S4A) than those produced by neutrophils from adult mice. Furthermore, macrophages isolated from the infant mice showed less killing capacity compared to the adult mice (Additional file 5: Figure S4B). These results suggest that ROS and NO from neutrophils are unlikely mediators of the more severe organ damage seen in the septic infant mice.

NETs have been implicated in organ dysfunction [23–25]. We therefore investigated the potential role of NETs in our sepsis model. Bone marrow neutrophils isolated from infant and adult mice were stimulated in vitro with PMA or LPS, and the formation of NETs was analyzed. We measured levels of NETs using myeloperoxidase-DNA complex as a specific marker for NETs [46]. Myeloperoxidase is a neutrophil-specific marker that can normally be detected in the neutrophil plasma. However, when NETosis occurs, myeloperoxidase is mixed with the neutrophil DNA, binding together when leaving the cell. Cells from infant mice produced markedly more NETs compared to that by the cells from adult mice (Fig. 2c, d; Additional file 5: Figure S4C, D). We then investigated NET production in vivo. Mice were injected with bacteria or LPS, and the concentration of NETs in the serum was analyzed 6 and 18 h later, respectively. Infant mice produced significantly more NETs than adult mice during LPS-induced endotoxemia or sepsis (Fig. 2e, f and Additional file 5: Figure S4E). The levels of NET clearance by serum DNase or macrophage phagocytosis from adult and infant mice were similar (Additional file 6: Figure S5A-D, and Additional file 7: Movie 1). These findings suggest that the high levels of NETs in the septic infant mice were due to the overwhelming NET production by neutrophils rather than a low level of NET clearance.


Additional file 7Movie 1 NET clearance by macrophages. (PDF 28600 KB) (MP4 28599 kb)


To confirm this hypothesis, we analyzed the expression of *Padi4* (peptidyl arginine deiminase 4), a neutrophil-enriched enzyme, which is critical in NET formation when chromatin is decondensed to form chromatin fibers [47]. An increase in histone citrullination is also associated with chromatin decondensation during NET formation [48, 49], and the conversion of histone arginine or monomethyl-arginine residue into citrulline by PAD4 [49, 50]. Neutrophils from infant mice expressed significantly more *Padi4* mRNA than the cells from adult mice when stimulated in vitro with PMA or LPS (Fig. 2g, h). Furthermore, circulating neutrophils from infant mice showed a significantly higher expression of *Padi4* than that of adult mice when injected with LPS (Fig. 2i). These differences were not due to variations in the frequency of circulating neutrophils (Additional file 8: Figure S6A-C).

### Degradation of NETs improves the outcome of sepsis

**Fig. 3:**
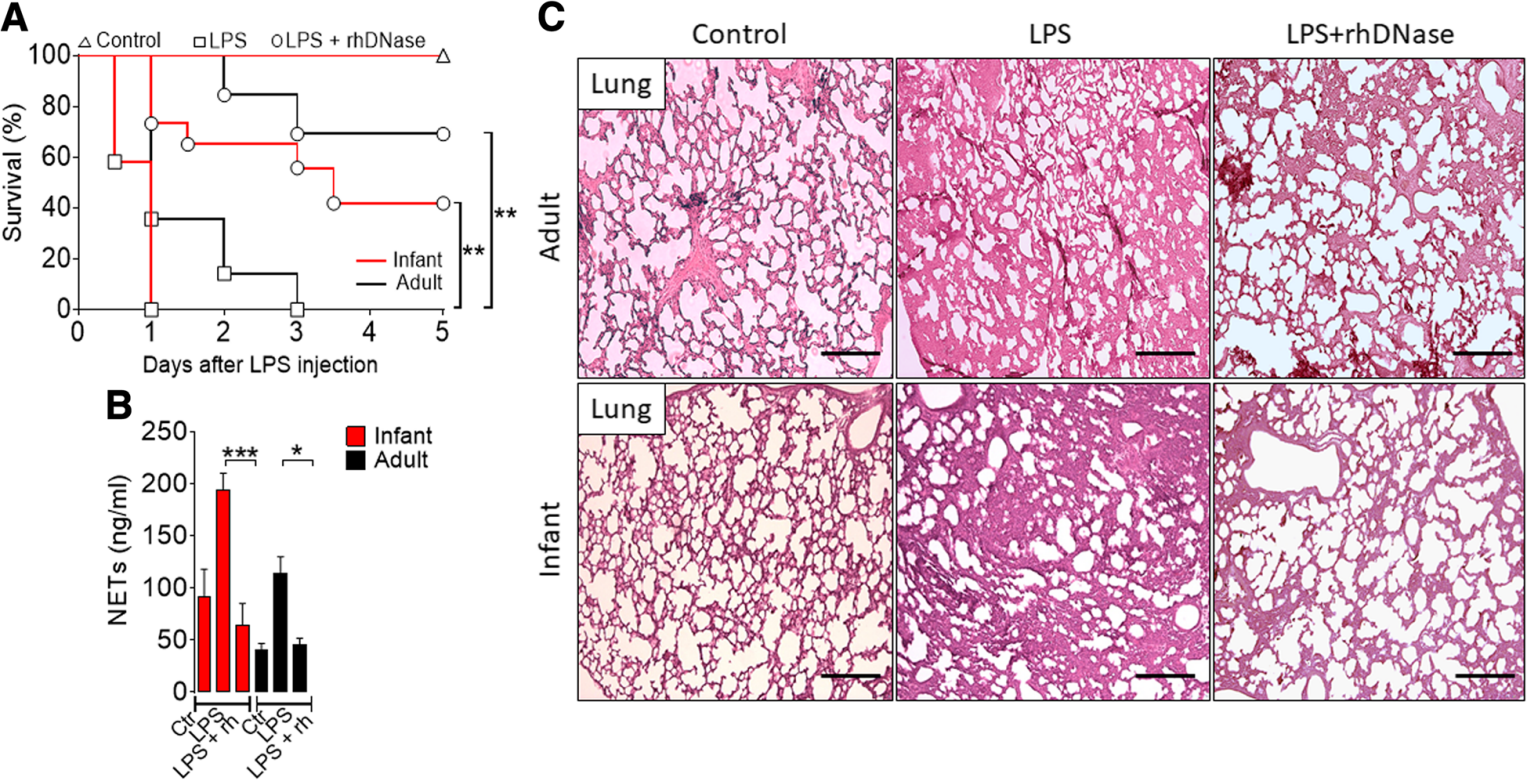
Degradation of NETs improves the outcome of LPS endotoxemia. **a** Survival of mice injected with LPS (35 mg/kg) and treated with saline (Ctr) or rhDNase (rh, 10 mg/kg, s.c.). **b** The concentration of NETs in the serum of mice 18 h after LPS injection and treated or not with rhDNase. **c** Representative histology of the lung from infant and adult mice 18 h after injection of LPS and with or without treatment of rhDNase, ×10 magnification. Bars = 50 μm. Data are mean ± SEM, *n* = 4–6, representative of two experiments, *p < 0.05, **p < 0.01, and ***p < 0.001 (**a** Mantel-Cox log-rank test; **b** one-way ANOVA, Bonferroni’s)

To investigate the functional role of NETs in the physiopathological context, a group of mice were injected with LPS and treated with rhDNase, a common treatment, to demonstrate the physiopathological role of NETs [51–53], which degrades DNA and NETs. LPS injection in both infant and adult mice led to high mortality, which was significantly reduced by the treatment with rhDNase (Fig. 3a). The higher survival rate in the groups treated with rhDNase was accompanied by a significant reduction of serum levels of NETs (Fig. 3b). The concentrations of BUN, CK-MB, and TNF-α in the serum were also significantly reduced by the treatment with rhDNase (Additional file 9: Figure S7A–C). Compared to untreated mice, histological analysis showed less septal edema, alveolar collapse, and vascular congestion in the lung of infant and adult LPS-injected mice treated with rhDNase (Fig. 3c). Together, these results suggest that elevated NET production could account for the higher susceptibility of infant mice to sepsis.

We extended this observation to the septic model induced by bacteria injection, treating the infant mice injected with bacteria with antibiotics (30 mg kg^− 1^ to adult mice and 15 mg kg^− 1^ to infant mice, beginning 6 h after CLP and then every 12 h up to day 3) plus rhDNase. As expected, antibiotics alone significantly reduced the mortality of the septic mice. The mortality was further reduced by the concomitant treatment with antibiotics plus rhDNase (Additional file 10: Figure S8A). The higher survival rate was accompanied by a significant reduction of serum NETs (Additional file 10: Figure S8B). Compared with the septic group treated with antibiotic alone, concentrations of BUN (biochemical markers of kidney damage) and GOT (liver injury) in the serum, and serum TNF-α were also significantly reduced by the treatment with antibiotic combined with rhDNase (Additional file 10: Figure S8C-E). Moreover, compared to that of the untreated or treated (only antibiotics) infant septic mice, histological analysis showed a marked reduction of edema and vascular congestion in the liver of infant septic mice treated with antibiotics plus rhDNase (Additional file 10: Figure S8F). It should be noted that treatment of the infant septic mice with rhDNase alone did not improve the survival rate (Additional file 11: Figure S9).

We then investigated the effect of the treatment of the septic infant mice with an inhibitor of PAD-4 (key enzyme involved in the NET formation) on the survival rate. It was observed that infant septic mice treated with the PAD4 inhibitor (Cl-Amidine) showed reduced mortality rate and this was further decreased with the combination therapy using antibiotic (Additional file 12: Figure S10). Together, these data suggest that the severe organ damage seen in the infant septic mice is likely due to the higher accumulation of neutrophils and elevated production of NETs by these organs.

### Degradation of NETs decreases histone citrullination in endotoxemic mice

**Fig. 4:**
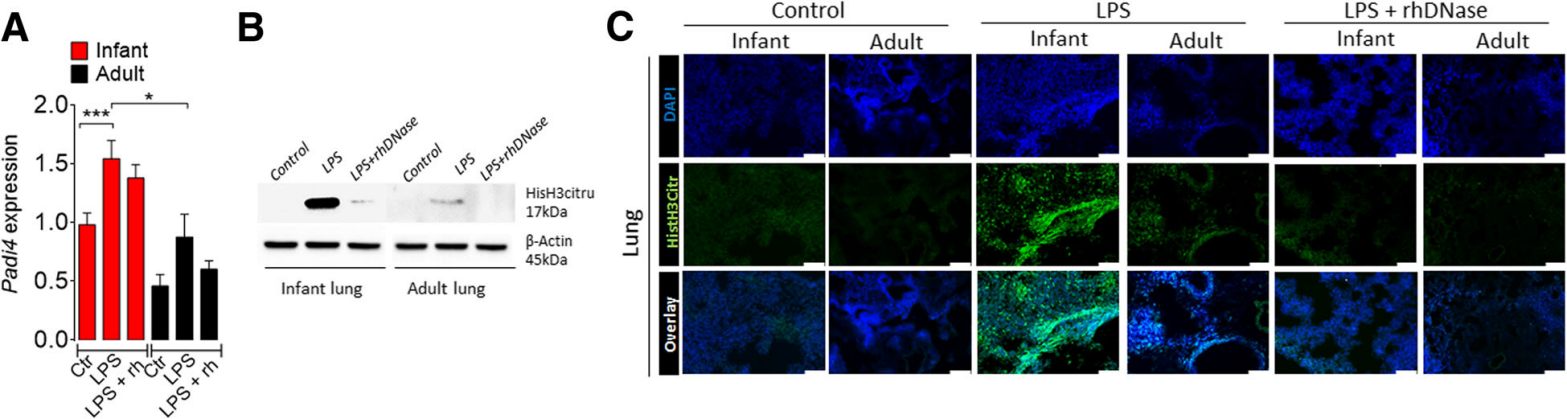
Degradation of NETs decreases histone citrullination in endotoxemic mice. **a** *Padi4* mRNA expression in the lung tissue from mice 18 h after LPS injection (35 mg/kg, i.p.) and treatment with or without rhDNase (10 mg/kg, s.c.). **b** Western blot and **c** representative images of immunofluorescence of histone H3 citrullination in the lungs of infant or adult mice 18 h after LPS injection. Bars = 50 μm. Data are mean ± SEM, *n* = 5–6 per group, ***p < 0.001 (one-way ANOVA, Bonferroni’s)

To further investigate the molecular mechanism involved in the increased release of NETs, we examined the expression of *Padi4* and histone H3 citrullination during rhDNase treatments. Infant and adult mice were injected with LPS and treated with rhDNase and their lungs and kidney harvested 18 h later. LPS markedly increased *Padi4* mRNA expression in both groups of mice. However, the increase of *Padi4* mRNA expression was not affected by the treatment of rhDNase (Fig. 4a), indicating that rhDNase acts downstream of *Padi4*. LPS injection significantly increased histone H3 citrullination in the lungs and kidney in the infant mice and, to a lesser extent, that of the adult mice (Fig. 4b, c and Additional file 13: Figure S11). These increases, as expected, were abolished by the treatment with rhDNase, suggesting that rhDNase directly degrades NETs.

### Serum NET concentration correlates with severity of pediatric sepsis

Finally, we assessed whether our data from the murine models could be extended to clinical sepsis. We used GEO datasets (GSE26378) of microarray gene files from pediatric patients performed within 24 h of pediatric intensive care unit admission compared with a group of healthy controls [54]. The gene set enrichment analysis (GSEA), with a focus on immunologic signature collection (immuneSigDB), shows that septic pediatric patients displayed a significant enrichment of genes related to LPS-stimulated neutrophils (Additional file 14: Figure S12A). Strikingly, a significant set of neutrophil activation genes, including *Padi4*, was upregulated in the septic pediatric patients (Additional file 14: Figure S12B). *Padi4* expression was also highly correlated with neutrophil activation markers, such as *S100A9* and *Il1b* [Spearman’s rank correlation coefficient (*r*) of 0.6168 and 0.2128, respectively] (Additional file 14: Figure S12C, D).

**Fig. 5:**
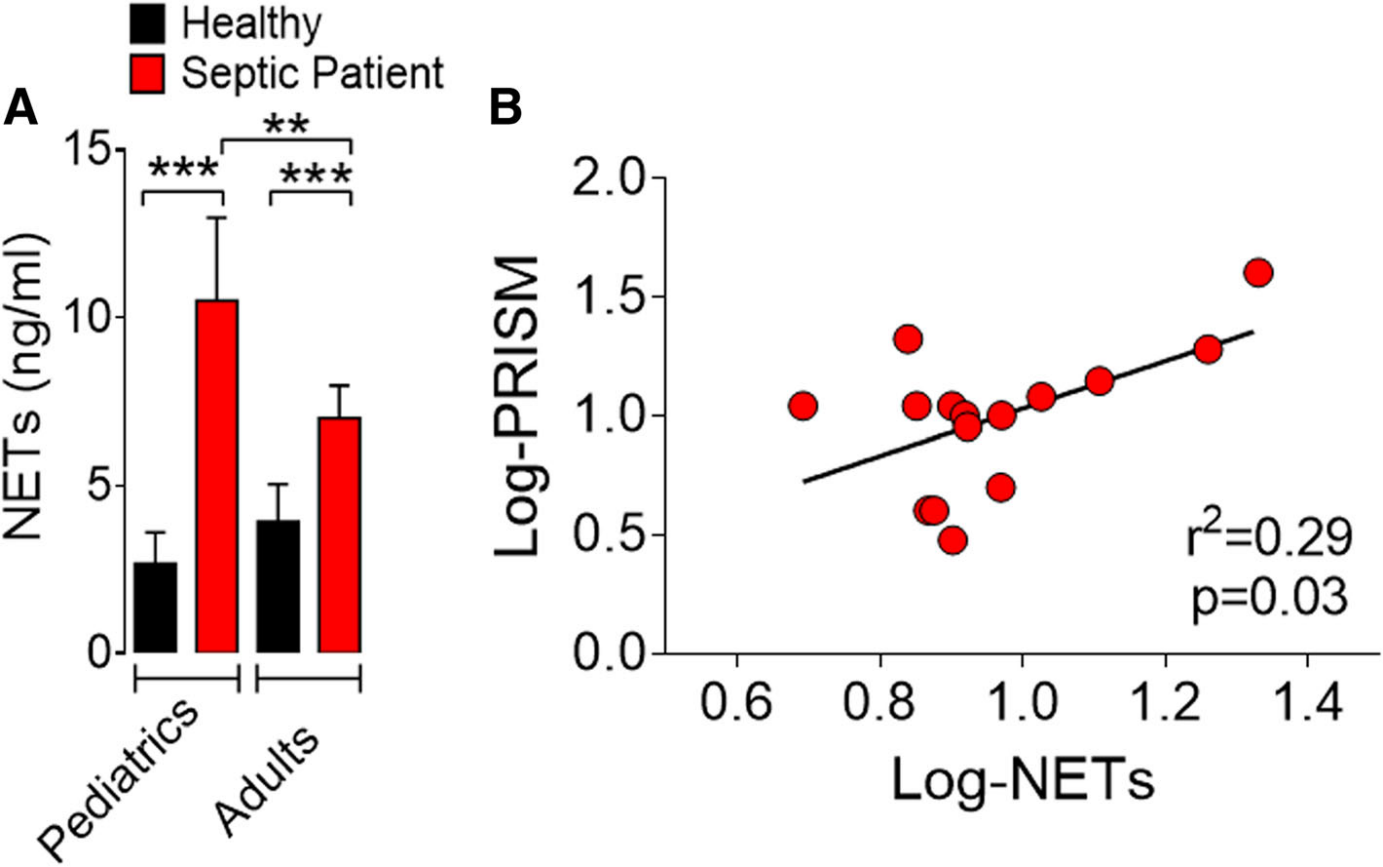
Serum NET concentration correlates with severity of pediatric sepsis. Blood samples were collected from pediatric and adult patients, and healthy volunteers, 24 h of hospital admission for sepsis. **a** NET concentrations in the serum of septic patients (15 pediatrics and 11 adults) and healthy individuals (7 pediatrics and 6 adults). **b** Correlation between serum Log-NET concentrations and Log-PRISM scores, **p < 0.01, ***p < 0.001 (**a** Kruskal-Wallis test and **b** Spearman correlation)

To confirm these in silico findings, we measured the serum concentrations of NETs in healthy control volunteers and septic patients. We prospectively included 26 patients (15 pediatrics and 11 adult septic patients) within the first 24 h of admission (t0) in the emergency department of a high-complexity hospital, and healthy volunteers (seven healthy children and six healthy adults) were included as controls. The baseline demographic and clinical characteristics are summarized in Additional file 15: Table S2. We recorded PRISM (Pediatric Risk of Mortality), PELOD (Pediatric Logistic Organ Risk of Mortality), and inotropic scores for pediatric patients and SOFA (Sequential Organ Failure Assessment) and APACHE II (Sepsis-Related Organ Failure Assessment) for adult patients. Most of the included patients were classified as having septic shock by the clinical staff. The clinical scores were log-transformed to approximate a normal distribution, which would render more reliable correlation results, eliminating any undue influence of outliers (see “Methods”). Compared to healthy control and adult septic patients, similar to the mice model results, pediatric septic patients showed higher serum levels of NETs (Fig. 5a). Pediatric septic patients exhibited significantly (*P* < 0.001) higher levels of *Padi4* expression in circulating neutrophils than that of the healthy controls, and there was a positive correlation between the level of serum NETs and the risk of mortality by sepsis in pediatric (Log-PRISM score) (Fig. 5b and Additional file 14: Figure S12E) and in adult patients [39].

## Discussion

Data reported here demonstrate that elevated levels of NETs are closely associated with the increased susceptibility of infant mice during sepsis compared to the adult mice. The high concentration of NETs could lead to exacerbated inflammatory response and multi-organ failure. The increased NET formation is linked to the increased expression of *Padi4* mRNA and histone H3 citrullination. Importantly, the pharmacological degradation of NETs or the inhibition of PAD-4 markedly attenuates the systemic inflammation and organ dysfunctions and improves the outcome of sepsis. In a translational manner, we demonstrated that infant sepsis patients exhibit higher concentrations of plasma NETs than adult sepsis patients and the level of NETs directly correlates with the severity of disease.

We used two models of sepsis in mice in the present study. Using a microbial injection, we found that infant mice were more susceptible than the adult mice, whether they were injected with the same number of bacteria or the equivalent number of bacteria per body weight. Similar results were obtained using an LPS model of sepsis. The infant mice injected with the same dose of LPS/kg body weight were more susceptible than adult mice. In the human context, the relative susceptibility of pediatric and adult patients to sepsis is controversial. Several reports showed that infant patients were more susceptible to sepsis [55, 56], while others found that the mortality rate in children was lower than that of adults [57–60]. This discrepancy is likely due to confounding factors such as co-morbidity and other concomitant infections in the heterogeneous human populations. Using inbred mice of defined breeding conditions, our data provide hitherto unrecognized observation that infant septic mice produced more NETs than adult septic mice. This increased NET production could account for the more severe outcome of infant sepsis. Importantly, pediatric septic patients also exhibit higher levels of NETs than healthy individuals, and the concentration of NETs correlates with the severity of the disease.

Conversely, it has been reported that PMNs isolated from cord blood derived from term and preterm infants fail to form NETs when activated with LPS or inflammatory agonists in the interval between 15 and 120 min [61]. The discrepancy is likely due to the heterogeneous source of the neutrophil populations and/or the elapse time of neutrophil stimulation. In fact, it was found that upon stimulation with LPS for 1 h, neonatal neutrophils did not show signs of NETosis; however, the process started after 2 h and reached the levels observed in adult neutrophils 3 h after LPS stimulation [62]. In the present study, we observed higher levels of NETosis at 4 h after in vitro neutrophil stimulation and 6 h after sepsis induction. Furthermore, compared with adults, the higher amounts of NETs produced by infant septic mice were accompanied with the increased expression of *Padi4* and hypercitrunillation of H3 histone. The NETosis is a highly controlled biological process and would result in cell death or vital NET release. The stimuli appear to be an important driver of these two processes. Stimuli, such as PMA or cholesterol crystals, induce late suicidal NETosis through a ROS-dependent mechanism [63], whereas complement receptors, TLR2/TLR4 ligands or TLR4-activated platelets, induce vital NETosis, through an ROS-independent mechanism. Moreover, both processes are dependent of PAD4 activation [47, 63]. Previous findings demonstrated that the *Pad4* expression and activity declines with age, peaking 2–4 weeks after birth and fell rapidly at 6–8 weeks old [64]. Concordantly, we observed that the infant *Pad4* expression baseline was higher compared to that of adults. Remarkably, we also observed that the PAD4 inhibitor, Cl-Amidine, alone decreased the mortality rate in septic animals and this was further reduced by the combination therapy with antibiotic. This result suggests that PAD4 inhibitors could also be used as a therapeutic option for pediatric sepsis.

To explore the potential of NETs as a target therapy for pediatric sepsis, infant animals were treated with rhDNase. It is noteworthy that rhDNase reduces the histone H3 citrullination, downstream of *Padi4* to prevent NETosis. It should also be noted that rhDNase alone (without antibiotics) was not able to attenuate sepsis-induced mortality in infants (Additional file 11: Figure S9). This susceptibility is most likely due to the overwhelming infection in the absence of antibiotics, since NETs are also an important microbicidal mediator [18, 39]. The more severe organ damage seen in the infant septic mice is likely due to the elevated production of inflammatory cytokines in these organs that promotes the accumulation of neutrophils, which produce NET and other cytotoxic mediators. Moreover, NETs can activate macrophages and dendritic cells to produce inflammatory cytokines including IL-1β, TNF-α, and IL-6 [65], which, apart from mediating organ damage, can also recruit more neutrophils to the end organs.

The clinical relevance of our finding was supported by the observation that the plasma of infant septic patients contained significantly higher concentrations of NETs than the adult septic patients and healthy controls. This is in accordance with previous report that described that the concentration of extracellular histones, an important component of NETs, is directly related to the severity of infant sepsis [66, 67]. Histones have been described as a mediator of endothelial dysfunction, organ failure, and death in septic patients [68]. However, it is important to point out that there is no clear consensus in the literature on the physiological equivalence by age between mice and humans. To mitigate this limitation, we performed a translational approach based on 2-week-old mice, which show similar patterns of immune responses to pediatric patients with a mean age of 3.5 years [56, 69, 70]. We observed that both mice and pediatric patients had an increase in the production of NETs during sepsis.

## Conclusion

Taking together, our findings demonstrate a potential use of serum NET levels as an indicator of sepsis severity. Moreover, pharmacological inhibition of NETs in combination with antibiotics represents a potential therapeutic strategy for the treatment of pediatric sepsis.

## Additional files

Additional file 1: **Figure S1.** Consort - The CONSORT diagram of the study. (PDF 734 kb)

Additional file 2: **Figure S2.** Standardization of lethal bacterial doses in infant and adult mice and in vitro NETs production by infant and adult neutrophils. (PDF 135 kb)

Additional file 3: **Figure S3.** Infant mice are more susceptible than adult mice to sepsis. (PDF 451 kb)

Additional file 4: **Table S1.** Infant mice exhibited more vascular hypo-responsiveness. (PDF 94 kb)

Additional file 5: **Figure S4.** NET production by infant and adult neutrophils. (PDF 240 kb)

Additional file 6: **Figure S5.** NETs clearance by serum DNase or macrophage phagocytosis from adult and infant mice are similar. (PDF 245 kb)

Additional file 8: **Figure S6.** (A) White Blood Cells (WBC) in infant and adult mice. Representative Flow Plots (B) and frequency (C) of spleen neutrophils (F4/80^−^Ly6G^+^ cells) in infant and adult mice. Results are mean ± SEM, *n* = 6–8 per group and are representative of 3 experiments. (PDF 59 KB). (PDF 58 kb)

Additional file 9: **Figure S7.** Degradation of NETs improves the outcome of LPS-induced endotoxemia. (PDF 125 kb)

Additional file 10: **Figure S8.** Degradation of NETs improves the outcome of sepsis. (PDF 291 kb)

Additional file 11: **Figure S9.** Degradation of NETs alone does not improve the outcome of infant sepsis. (PDF 22 kb)

Additional file 12: **Figure S10.** Inhibition of PAD4 improves the outcome of sepsis. (PDF 17 kb)

Additional file 13: **Figure S11.** Degradation of NETs decreases histone citrullination in endotoxemic mice. (PDF 174 kb)

Additional file 14: **Figure S12.** Neutrophil activation and *Padi4* expression on pediatric septic patients. (PDF 617 kb)

Additional file 15: **Table S2.** Baseline demographic and clinical characteristics of the septic patients. SOFA, Sequential Organ Failure Assessment. APACHE, Acute Physiology and Chronic Health Evaluation. PRISM, Pediatric Risk of Mortality. PELOD, Pediatric Logistic Organ Dysfunction. PRISM, Pediatric Risk of Mortality. (PDF 192 KB). (PDF 191 kb)

## Abbreviations

BUN: Blood urea nitrogen
CK-MB: Creatine kinase-MB isoenzyme
GOT: Glutamic oxaloacetic transaminase
NETs: Neutrophil extracellular traps
NOS: Nitrogen species
Padi4: Peptidyl arginine deiminase 4
rh: Recombinant human
ROS: Reactive oxygen species
TLR: Toll-like receptor
