# Supplementary figures and images for: Neutrophil extracellular traps (NETs) exacerbate severity of infant sepsis
Source: Crit Care. 2019 Apr 8;23:113. doi: 10.1186/s13054-019-2407-8 (PMC6454713; doi:10.1186/s13054-019-2407-8)

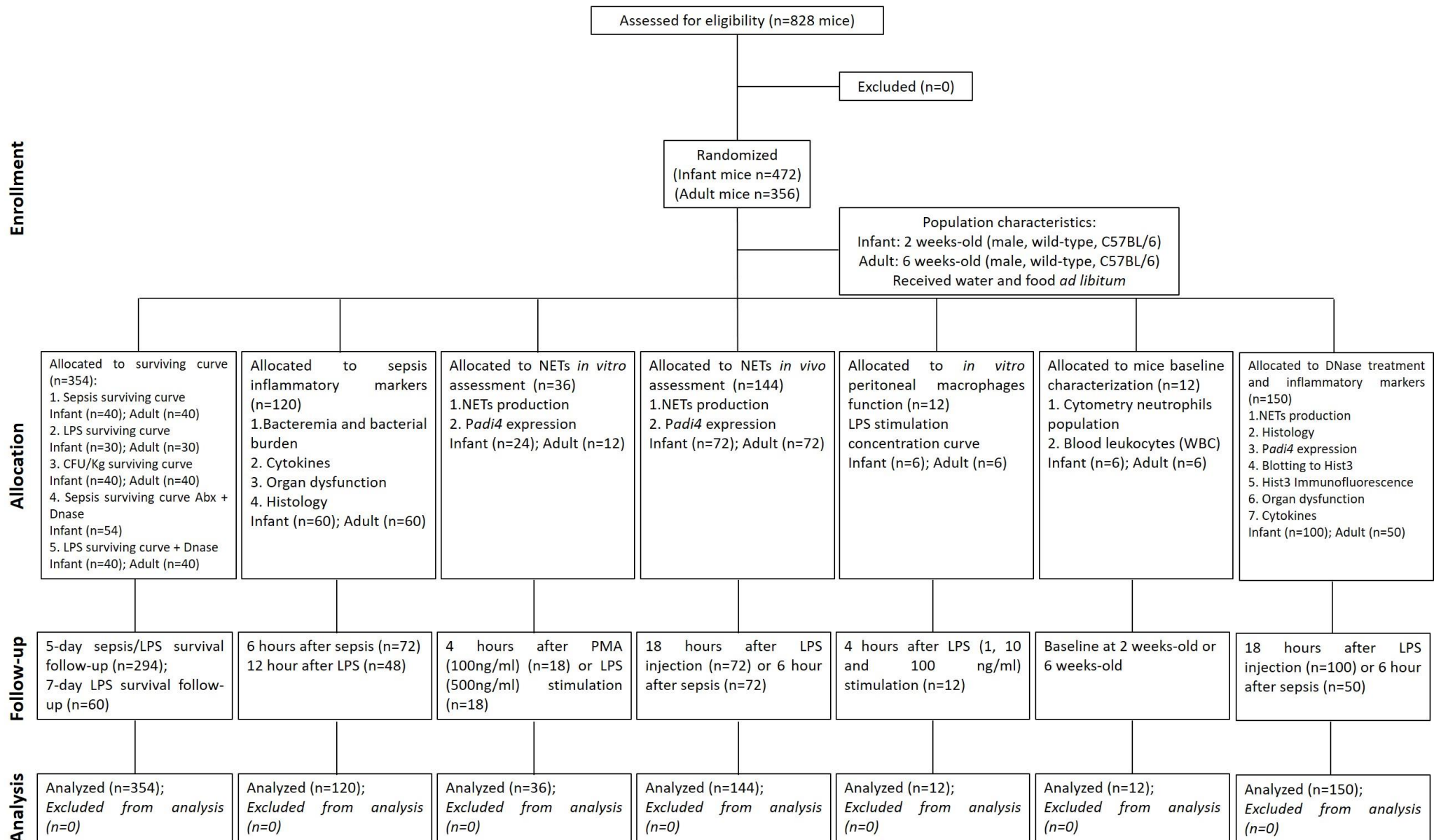

**FIGURE S1. CONSORT diagram of the study.**

Supplement: Supplementary file 2 — Figure S1. Consort - The CONSORT diagram of the study. (PDF 734 kb) [file 13054_2019_2407_MOESM1_ESM.pdf]
