# Supplementary material for: Neutrophil extracellular traps (NETs) exacerbate severity of infant sepsis
Source: Crit Care. 2019 Apr 8;23:113. doi: 10.1186/s13054-019-2407-8 (PMC6454713; doi:10.1186/s13054-019-2407-8)
Supplement: Supplementary file 3 — Figure S2. Standardization of lethal bacterial doses in infant and adult mice and in vitro NETs production by infant and adult neutrophils. (PDF 135 kb) [file 13054_2019_2407_MOESM2_ESM.pdf]

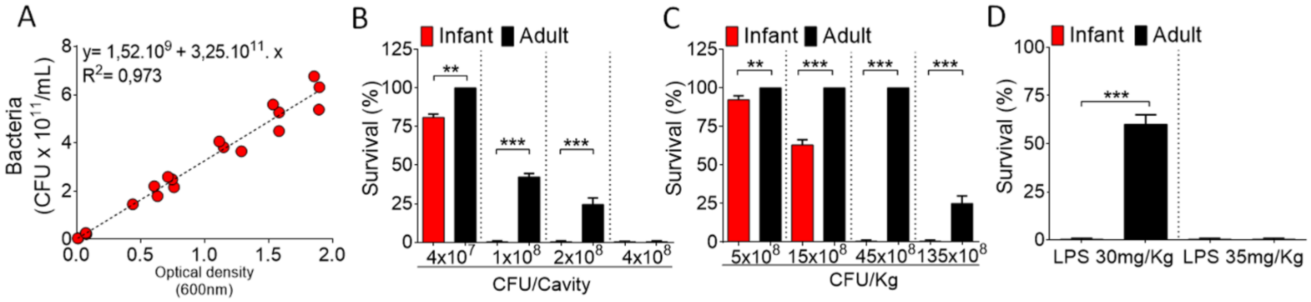

FIGURE S2. Standardization of lethal bacterial doses in infant and adult mice and *in vitro* NETs production by infant and adult neutrophils. **(A)** Number of bacteria calculated according optical density on 600 nm and culture on Mueller-Hinton agar plates**.** Infant and adult mice were injected intraperitoneally with graded doses of polymicrobial bacterial suspension in CFU/cavity **(B)** or CFU/kg body weight **(C)**. In separate experiments, mice were injected i.p. with graded dose of LPS (mg/kg; **D**). The survival rates were recorded for 5 days. n=5-6 per group, *p<0.05, ** p<0.01, *** p<0.001 (Mantel-Cox log-rank test). On average, the infant mice were 10 grams and the adult mice 20 grams in body weight. Note that there was 100% mortality rate when the infant mice were injected with doses higher than 1 x10^8 bacteria. However, for adult mice, the 100% mortality was observed only with doses higher than 4 x x10^8 bacteria.
