# Supplementary material for: Neutrophil extracellular traps (NETs) exacerbate severity of infant sepsis
Source: Crit Care. 2019 Apr 8;23:113. doi: 10.1186/s13054-019-2407-8 (PMC6454713; doi:10.1186/s13054-019-2407-8)
Supplement: Supplementary file 4 — Figure S3. Infant mice are more susceptible than adult mice to sepsis. (PDF 451 kb) [file 13054_2019_2407_MOESM3_ESM.pdf]

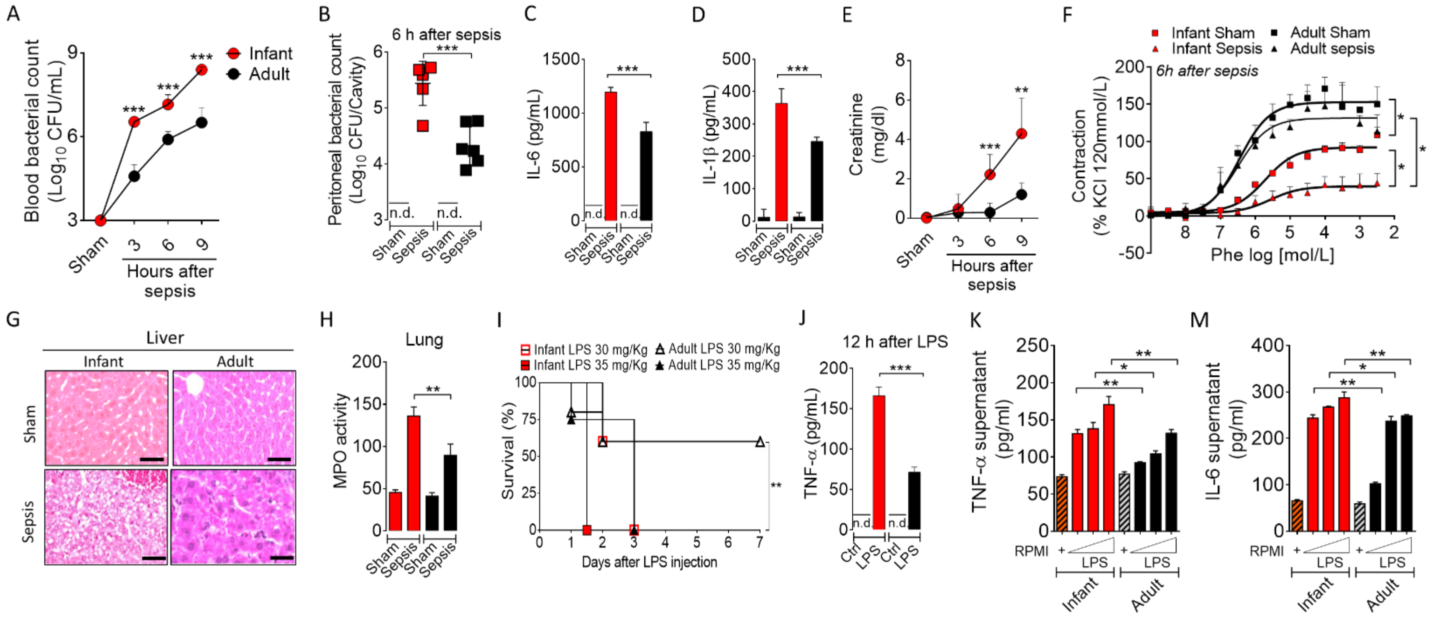

FIGURE S3. Infant mice are more susceptible than adult mice to sepsis. Infant mice are more susceptible than adult mice to sepsis. Infant and adult mice were injected with microbial suspension. Bacterial counts in the blood **(A)** and peritoneal cavity **(B)**. Serum concentrations of IL-6 **(C)** and IL-1β **(D)** were determined by ELISA 6 h after sepsis induction. Serum concentrations of creatinine **(E)** were determined after sepsis. Vascular reactivity **(F)** and representative histology **(G)** of liver sections are shown, magnification 40x. Bars = 100 µm. **(H)** Activity of MPO in the lung (measure of neutrophil infiltration) was determined. **(I)** Survival of mice injected with LPS. Serum concentrations of TNF-α **(J)** were determined by ELISA 12 h after LPS injection. Peritoneal macrophages were cultured with LPS (1, 10 and 100 ng/ml) for 4 h, and the concentrations of TNF-α **(K)** and IL-6 **(M)** in the culture supernatant were determined by ELISA. Data are mean ± SEM, n=5-6 per group and are representative of 2-3 independent experiments. Ctr = Control group, Sep = Sepsis group, *p<0.05, ** p<0.01, *** p<0.001. (**A – E, H, J – M**, one way-ANOVA, Bonferroni's; **F**, % of maximum response, one way-ANOVA, **I**, Mantel-Cox log-rank test).
