# Supplementary material for: Neutrophil extracellular traps (NETs) exacerbate severity of infant sepsis
Source: Crit Care. 2019 Apr 8;23:113. doi: 10.1186/s13054-019-2407-8 (PMC6454713; doi:10.1186/s13054-019-2407-8)
Supplement: Supplementary file 5 — Table S1. Infant mice exhibited more vascular hypo-responsiveness. (PDF 94 kb) [file 13054_2019_2407_MOESM4_ESM.pdf]

TABLE S1 – Infant mice exhibited more vascular hypo-responsiveness.

Groups	pD_2 values 6 h
**Infant Sham**	5.84 ± 0.03
**Infant Sepsis**	5.17 ± 0.33[#,*]
**Adult Sham**	6.85 ± 0.08
**Adult Sepsis**	6.2 ± 0.14[&]

pD_2 of concentration-response curve to phenylephrine (10-10-10-5 mol/L) 6 h after sepsis. Data are the mean ± SEM, n=5-6, representative of two experiments, #p<0.05 Infant septic vs. Infant Sham; &p<0.05 Adult Septic vs. Adult Sham; *p<0.05 Infant Septic vs. Adult Septic (% of maximum response, one way-ANOVA, Bonferroni's).

* pD2 = -log (EC_{50}), EC_{50}: Half maximal effective concentration.
