# Supplementary material for: Neutrophil extracellular traps (NETs) exacerbate severity of infant sepsis
Source: Crit Care. 2019 Apr 8;23:113. doi: 10.1186/s13054-019-2407-8 (PMC6454713; doi:10.1186/s13054-019-2407-8)
Supplement: Supplementary file 6 — Figure S4. NET production by infant and adult neutrophils. (PDF 240 kb) [file 13054_2019_2407_MOESM5_ESM.pdf]

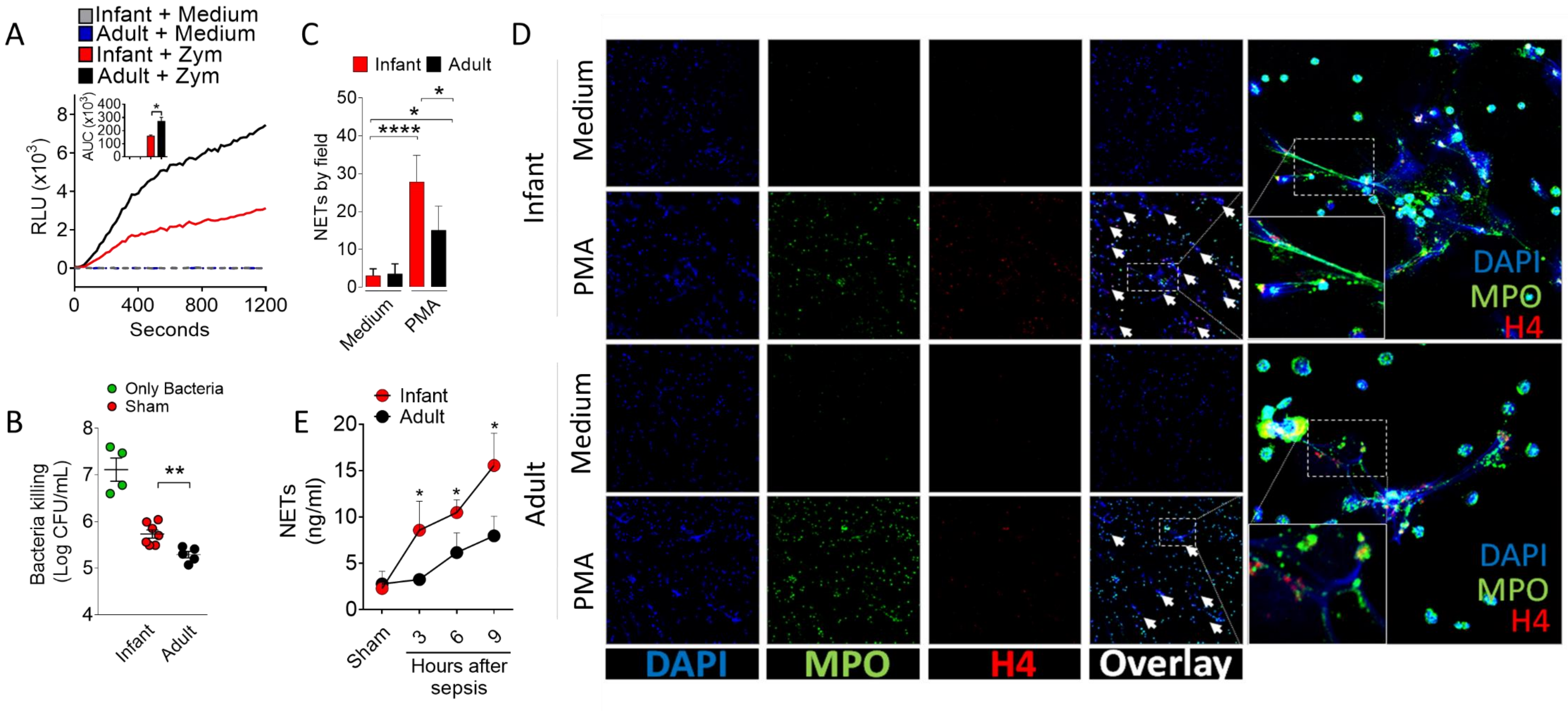

FIGURE S4. NET production by infant and adult neutrophils. **(A)** Bone marrow-isolated neutrophils from mice were cultured with zymosan (20 mg/ml) for 20 min and the production of ROS determined. **(B)** Peritoneal cavity macrophages (1 x 10^6) from adult and infant were collected and incubated with bacteria of the cecal content (2 x 10^6) previously opsonized with 10% serum and incubated for 3 h at 37°C. After incubation, the cells were lysed and seeded at the 10^{-4} dilution. **(C-D)** Bone Marrow neutrophils (1 x 10^6) from infant and adult mice were stimulated in vitro with PMA (100 ng/mL) for 4 h. Representative immunofluorescence images of NETs released by neutrophils and quantitation of NETs by field. **(E)** NETs concentration in the serum was determined 3, 6 and 9 h after sepsis induction. Results are presented as the mean ± SEM, n = 6-8 per group and are representative of 3 experiments. Phagocytic killing is presented as log of UFC/mL. * $p < 0.05$ and ** $p < 0.01$.
