# Supplementary material for: Neutrophil extracellular traps (NETs) exacerbate severity of infant sepsis
Source: Crit Care. 2019 Apr 8;23:113. doi: 10.1186/s13054-019-2407-8 (PMC6454713; doi:10.1186/s13054-019-2407-8)
Supplement: Supplementary file 7 — Figure S5. NETs clearance by serum DNase or macrophage phagocytosis from adult and infant mice are similar. (PDF 245 kb) [file 13054_2019_2407_MOESM6_ESM.pdf]

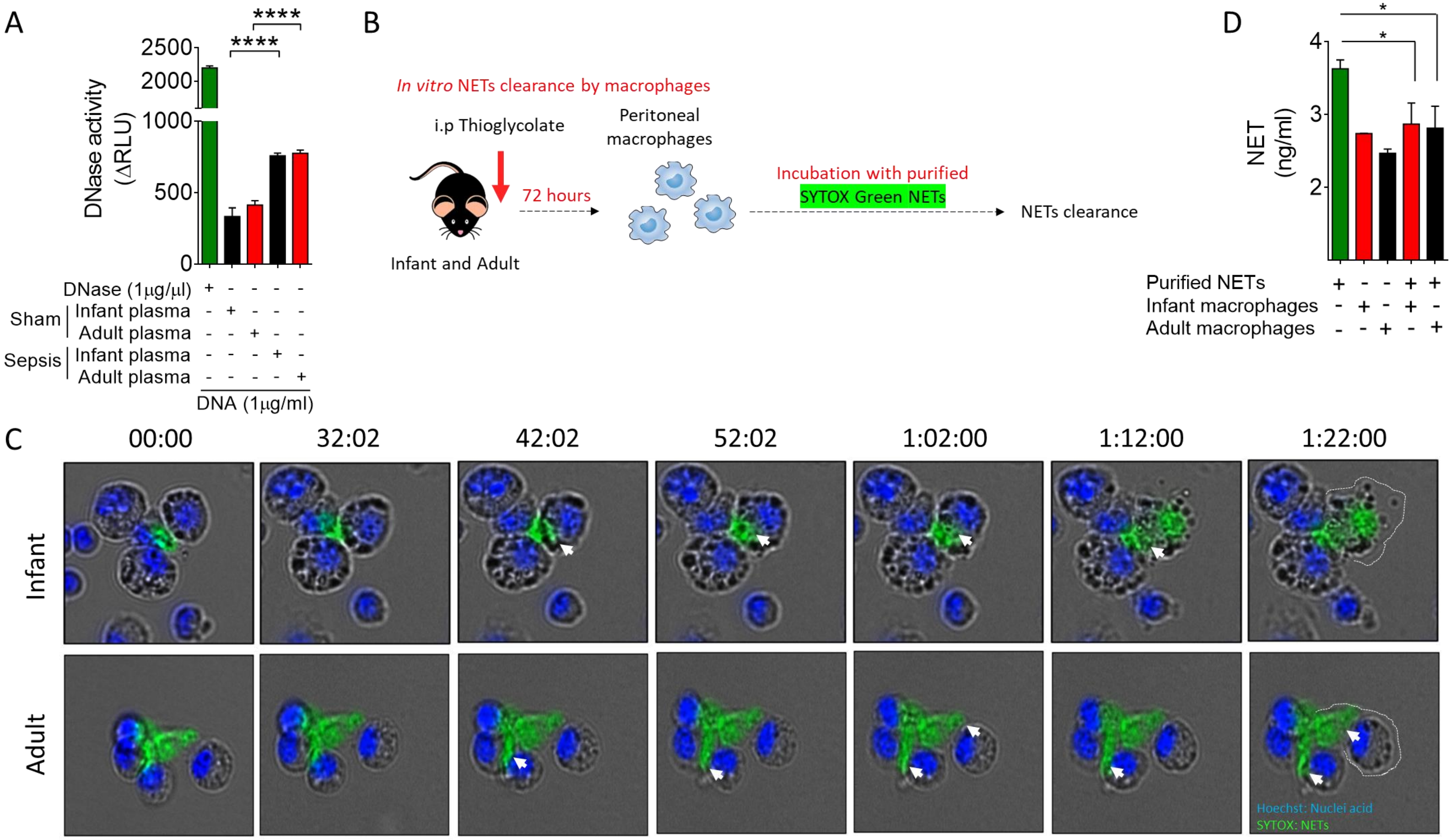

FIGURE S5. The levels of NETs clearance by serum DNase or macrophage phagocytosis from adult and infant mice are similar. Animals undergoing sepsis were euthanized 6 h after infection and blood was collected. **(A)** DNase plasma activity 6 h after sepsis. **(B)** Protocol of NETs macrophages clearance. **(C)** Representative time course of SITOX-green NETs macrophages clearance. **(D)** Remaining NETs levels in the culture supernatants of macrophages and purified NETs (4 ng/mL) after 4 h. Results are mean ± SEM, n = 6-8 per group and are representative of 2 experiments. * p <0.05, ** p <0.01 and **** p <0.0001.
