# Supplementary material for: Neutrophil extracellular traps (NETs) exacerbate severity of infant sepsis
Source: Crit Care. 2019 Apr 8;23:113. doi: 10.1186/s13054-019-2407-8 (PMC6454713; doi:10.1186/s13054-019-2407-8)
Supplement: Supplementary file 8 — Figure S6. (A) White Blood Cells (WBC) in infant and adult mice. Representative Flow Plots (B) and frequency (C) of spleen neutrophils (F4/80−Ly6G+ cells) in infant and adult mice. Results are mean ± SEM, n = 6–8 per group and are representative of 3 experiments. (PDF 59 KB). (PDF 58 kb) [file 13054_2019_2407_MOESM8_ESM.pdf]

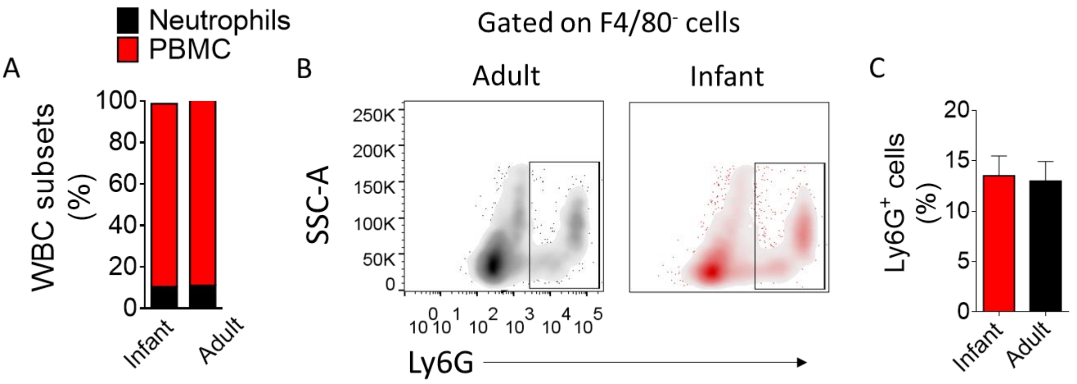

FIGURE S6. (A) White Blood Cells (WBC) in infant and adult mice. **(B-C)** Frequency and representative Flow Plots of spleen neutrophils (F4/80-Ly6G+ cells) in infant and adult mice. Results are mean ± SEM, n = 6-8 per group and are representative of 3 experiments.
