# Supplementary material for: Neutrophil extracellular traps (NETs) exacerbate severity of infant sepsis
Source: Crit Care. 2019 Apr 8;23:113. doi: 10.1186/s13054-019-2407-8 (PMC6454713; doi:10.1186/s13054-019-2407-8)
Supplement: Supplementary file 9 — Figure S7. Degradation of NETs improves the outcome of LPS-induced endotoxemia. (PDF 125 kb) [file 13054_2019_2407_MOESM9_ESM.pdf]

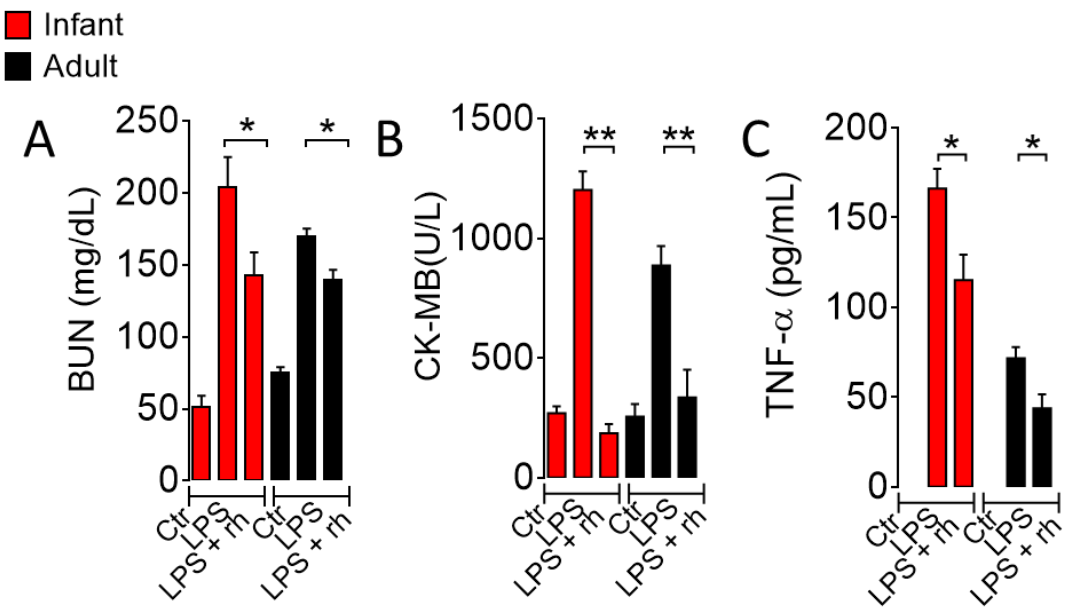

FIGURE S7. Degradation of NETs improves the outcome of LPS endotoxemia. (A) BUN, CK-MB **(B)** and TNF-α **(C)** in the serum of mice 18 h after LPS injection and treated or not with rhDNase (rh). Data are mean ± SEM, n=5-6, representative of two experiments, *$p<0.05$ and ** $p<0.01$ (one way-ANOVA, Bonferroni's).
