# Supplementary material for: Neutrophil extracellular traps (NETs) exacerbate severity of infant sepsis
Source: Crit Care. 2019 Apr 8;23:113. doi: 10.1186/s13054-019-2407-8 (PMC6454713; doi:10.1186/s13054-019-2407-8)
Supplement: Supplementary file 10 — Figure S8. Degradation of NETs improves the outcome of sepsis. (PDF 291 kb) [file 13054_2019_2407_MOESM10_ESM.pdf]

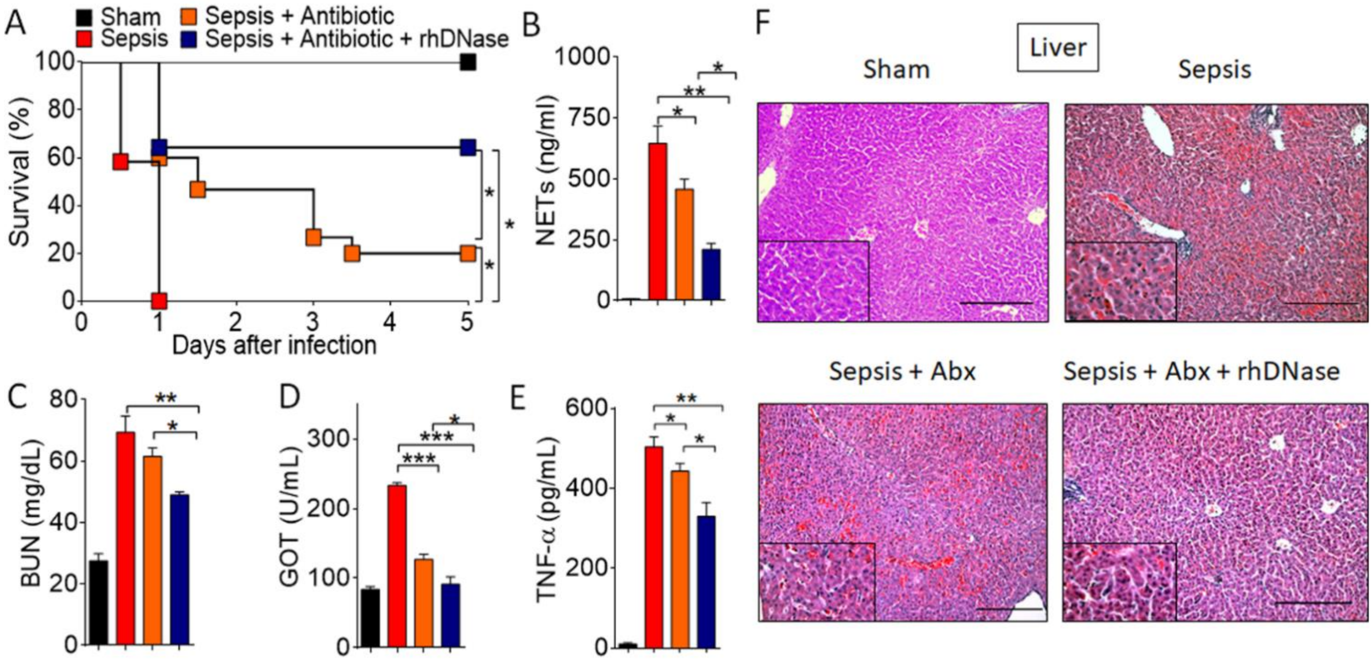

FIGURE S8. Degradation of NETs improves the outcome of sepsis. (A) Survival of infant septic mice treated with saline, antibiotics (Abx) alone, or Abx + rhDNase (10 mg/kg s.c.). **(B)** NETs, **(C)** BUN, **(D)** GOT and **(E)** TNF-α levels in the serum 6 h after sepsis induction were measured. **(F)** Representative liver sections from the indicated groups were stained with hematoxylin/eosin for histological examination, 20x and 40x magnification. Bars = 100 µm. Data are mean ± SEM, n=5-6, representative of two experiments, *$p<0.05$, ** $p<0.01$ and *** $p<0.001$ (A, Mantel-Cox log-rank test; B – E, one way-ANOVA, Bonferroni's).
