# Supplementary material for: Neutrophil extracellular traps (NETs) exacerbate severity of infant sepsis
Source: Crit Care. 2019 Apr 8;23:113. doi: 10.1186/s13054-019-2407-8 (PMC6454713; doi:10.1186/s13054-019-2407-8)
Supplement: Supplementary file 11 — Figure S9. Degradation of NETs alone does not improve the outcome of infant sepsis. (PDF 22 kb) [file 13054_2019_2407_MOESM11_ESM.pdf]

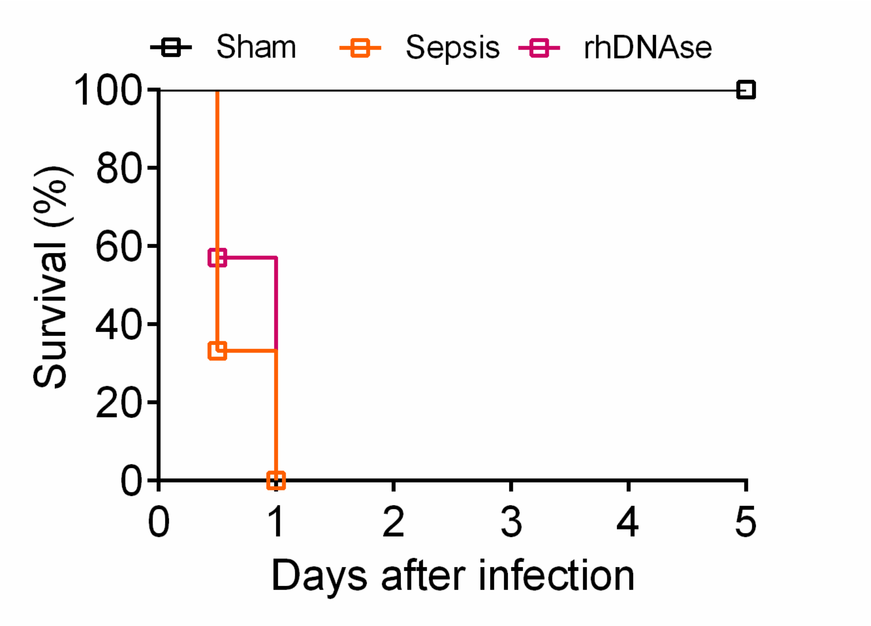

FIGURE S9. Degradation of NETs alone does not improve the outcome of infant sepsis. The survival rates were evaluated during 5 days in bacteria-injected mice ($2x10^8$ CFU) treated with saline or rhDNase (10 mg/kg s.c.). n=5-6, representative of two experiments.
