# Supplementary material for: Neutrophil extracellular traps (NETs) exacerbate severity of infant sepsis
Source: Crit Care. 2019 Apr 8;23:113. doi: 10.1186/s13054-019-2407-8 (PMC6454713; doi:10.1186/s13054-019-2407-8)
Supplement: Supplementary file 12 — Figure S10. Inhibition of PAD4 improves the outcome of sepsis. (PDF 17 kb) [file 13054_2019_2407_MOESM12_ESM.pdf]

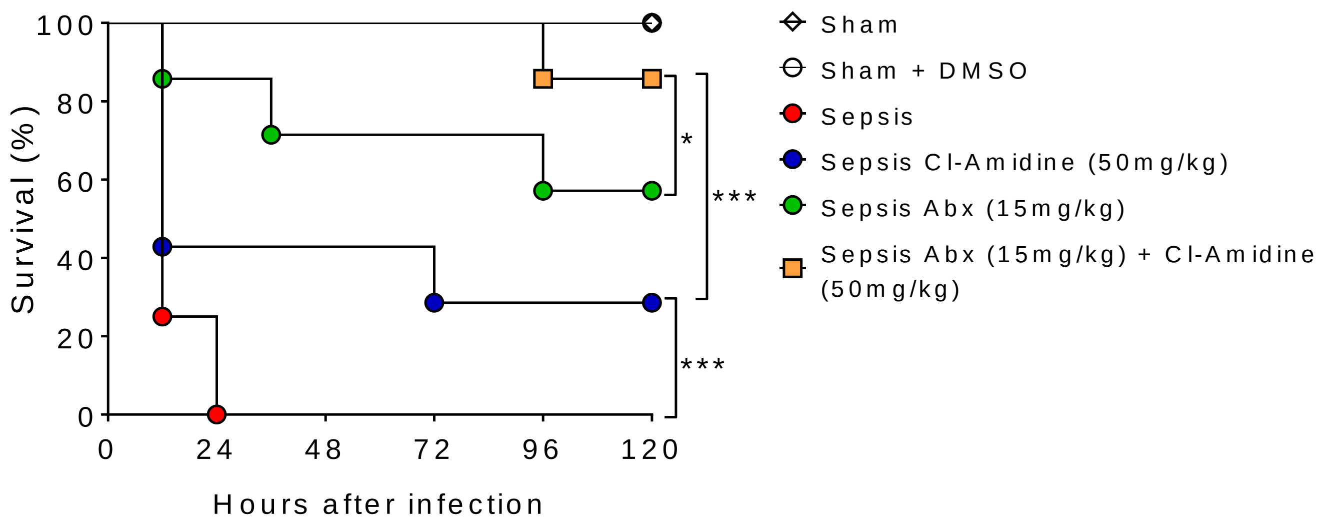

FIGURE S10. Inhibition of PAD4 improves the outcome of sepsis. Infant mice were injected with microbial suspension and treated i.p with Cl-amidine (PAD4 inhibitor) 50 mg/kg 1 h before sepsis with or without antibiotic association. n=5-6. * $p<0.05$ and *** $p<0.001$ (Mantel-Cox log-rank test).
