# Supplementary material for: Neutrophil extracellular traps (NETs) exacerbate severity of infant sepsis
Source: Crit Care. 2019 Apr 8;23:113. doi: 10.1186/s13054-019-2407-8 (PMC6454713; doi:10.1186/s13054-019-2407-8)
Supplement: Supplementary file 13 — Figure S11. Degradation of NETs decreases histone citrullination in endotoxemic mice. (PDF 174 kb) [file 13054_2019_2407_MOESM13_ESM.pdf]

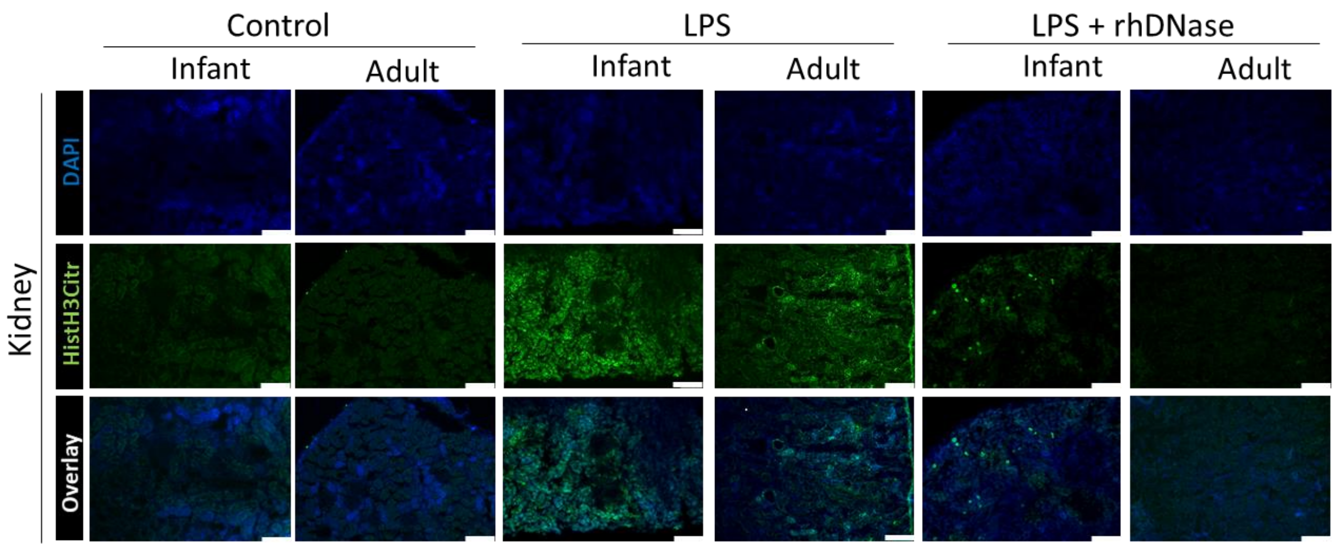

FIGURE S11. Degradation of NETs decreases histone citrullination in endotoxemic mice. Representative images of immunofluorescence of histone H3 citrullination in the kidney of infant or adult mice 18 h after LPS injection. Bars = 50 µm. Data are mean ± SEM, n=5-6 per group.
