# Supplementary material for: Neutrophil extracellular traps (NETs) exacerbate severity of infant sepsis
Source: Crit Care. 2019 Apr 8;23:113. doi: 10.1186/s13054-019-2407-8 (PMC6454713; doi:10.1186/s13054-019-2407-8)
Supplement: Supplementary file 14 — Figure S12. Neutrophil activation and Padi4 expression on pediatric septic patients. (PDF 617 kb) [file 13054_2019_2407_MOESM14_ESM.pdf]

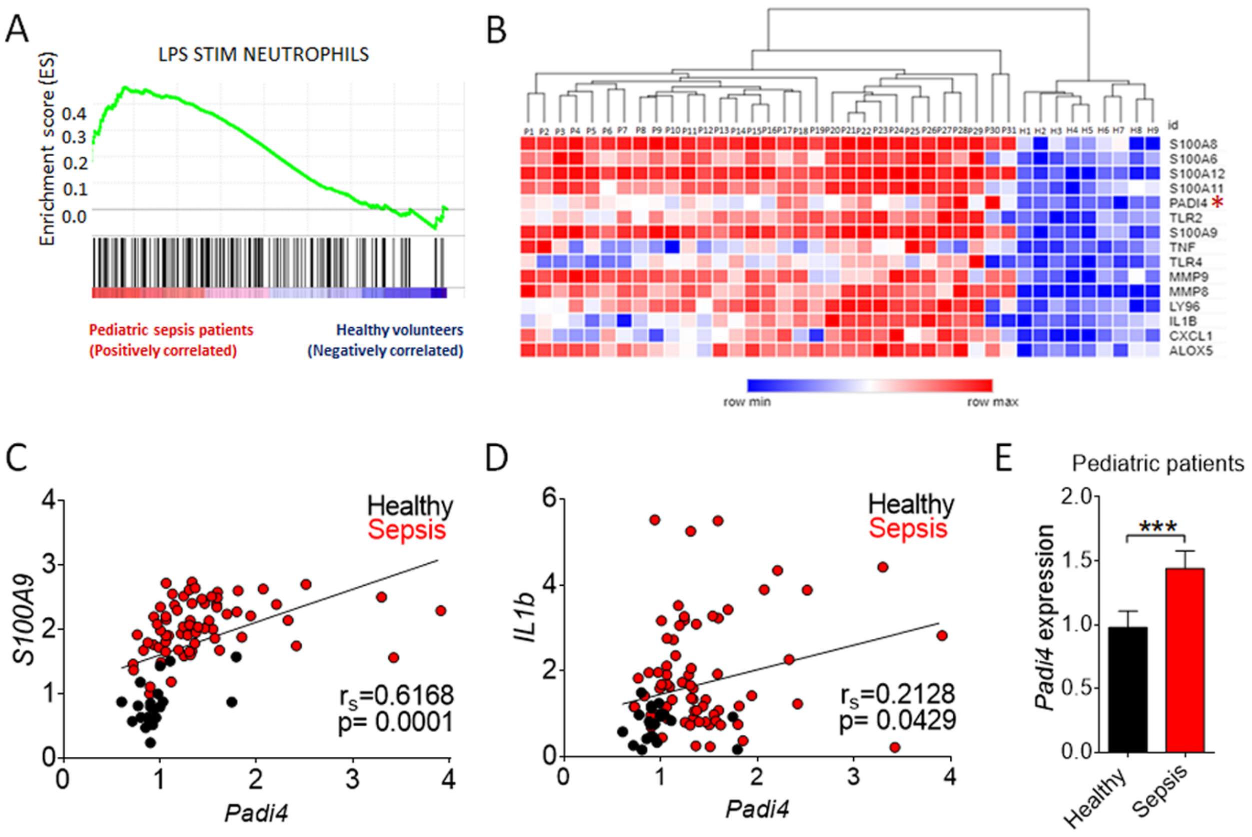

FIGURE S12. Neutrophil activation and *Padi4* expression on pediatric septic patients. Gene set enrichment analysis (GSEA) from a public expression-profiling array (GSE26378) of pediatric patients within 24 h of hospital admission compared to a group of healthy controls. **(A)** Enrichment of genes related to activated neutrophil enrichment plot from GSEA analysis. The green curve shows the running enrichment score (ES) for the gene set as the analysis walks down the ranked gene list, the leading edge for the gene set is shown as short bars at the bottom. **(B)** Heatmap shows the most representative genes related to activated neutrophils from GSEA analysis and among them *Padi4* (*). **(C-D)** Spearman's rank correlation coefficient between *Padi4* expression and neutrophil activation markers *S100A9* and *IL-1β*. Blood samples were collected from pediatric and adult patients, and healthy volunteers, 24 h of hospital admission for sepsis. **(E)** *Padi4* mRNA expression in neutrophils from pediatric sepsis patients (n=15) and healthy controls (n=7).
