# Supplementary material for: Neutrophil extracellular traps (NETs) exacerbate severity of infant sepsis
Source: Crit Care. 2019 Apr 8;23:113. doi: 10.1186/s13054-019-2407-8 (PMC6454713; doi:10.1186/s13054-019-2407-8)
Supplement: Supplementary file 15 — Table S2. Baseline demographic and clinical characteristics of the septic patients. SOFA, Sequential Organ Failure Assessment. APACHE, Acute Physiology and Chronic Health Evaluation. PRISM, Pediatric Risk of Mortality. PELOD, Pediatric Logistic Organ Dysfunction. PRISM, Pediatric Risk of Mortality. (PDF 192 KB). (PDF 191 kb) [file 13054_2019_2407_MOESM15_ESM.pdf]

TABLE S2 – Baseline demographic and clinical characteristics of the septic patients.

Characteristics	Adult Patients (n=11)	Pediatric Patients (n=15)
**Age (years) – Median (SEM)**	60.33 (±22)	3.52 (±5.07)
**Female – n (%)**	4 (28.5)	8 (53.3)
**APACHE II – median (SEM)**	13.8 (±6.91)	N/A
**SOFA – mean (SEM)**	6.93 (±3.30)	N/A
**PRISM – mean (SEM)**	N/A	9.18 (±4.68)
**PELOD – mean (SEM)**	N/A	9.45 (±6.48)
**Inotropic score**	N/A	20.8 (±17.21)

N/A – Non-applied

SOFA, Sequential Organ Failure Assessment. APACHE, Acute Physiology and Chronic Health Evaluation. PRISM, Pediatric Risk of Mortality. PELOD, Pediatric Logistic Organ Dysfunction. PRISM, Pediatric Risk of Mortality.
